# Supplementary material for: Antifungal alternation can be beneficial for durability but at the cost of generalist resistance
Source: Commun Biol. 2023 Feb 16;6:180. doi: 10.1038/s42003-023-04550-6 (PMC9935548; doi:10.1038/s42003-023-04550-6)
Supplement: Supplementary file 2 — Supplementary Material [file 42003_2023_4550_MOESM2_ESM.pdf]

Supplementary Information

Antifungal alternation can be beneficial for durability but at the cost of generalist resistance

Agathe Ballu[1], Philomène Despréaux[1], Clémentine Duplaix[1], Anne Dérédec[1], Florence Carpentier[2,3¶], Anne-Sophie Walker[1*¶]

[1]Université Paris-Saclay, INRAE, AgroParisTech, UMR BIOGER, 78850 Thiverval-Grignon, France

[2]Université Paris-Saclay, INRAE, UR MaIAGE, 78350 Jouy-en-Josas, France

[3]AgroParisTech, 75005 Paris, France

[¶]These authors contributed equally to this work

*Materials and correspondence requests should be addressed to Dr Anne-Sophie Walker ✉ anne-sophie.walker@inrae.fr

Supplementary Information Figure S1: Variation of resistance evolution in selection regimes

ρ, the mean resistance evolution rate, is shown for each selection regime. The error bars represent the standard error *P*-values for pairwise comparisons were obtained with linear models (Tukey's *post-hoc* correction). Alternations involving B, C or P had a beneficial or neutral effect on resistance evolution rate relative to the continuous use of the same fungicide. The benefit of alternation depends on the intrinsic resistance risks of the paired AIs.

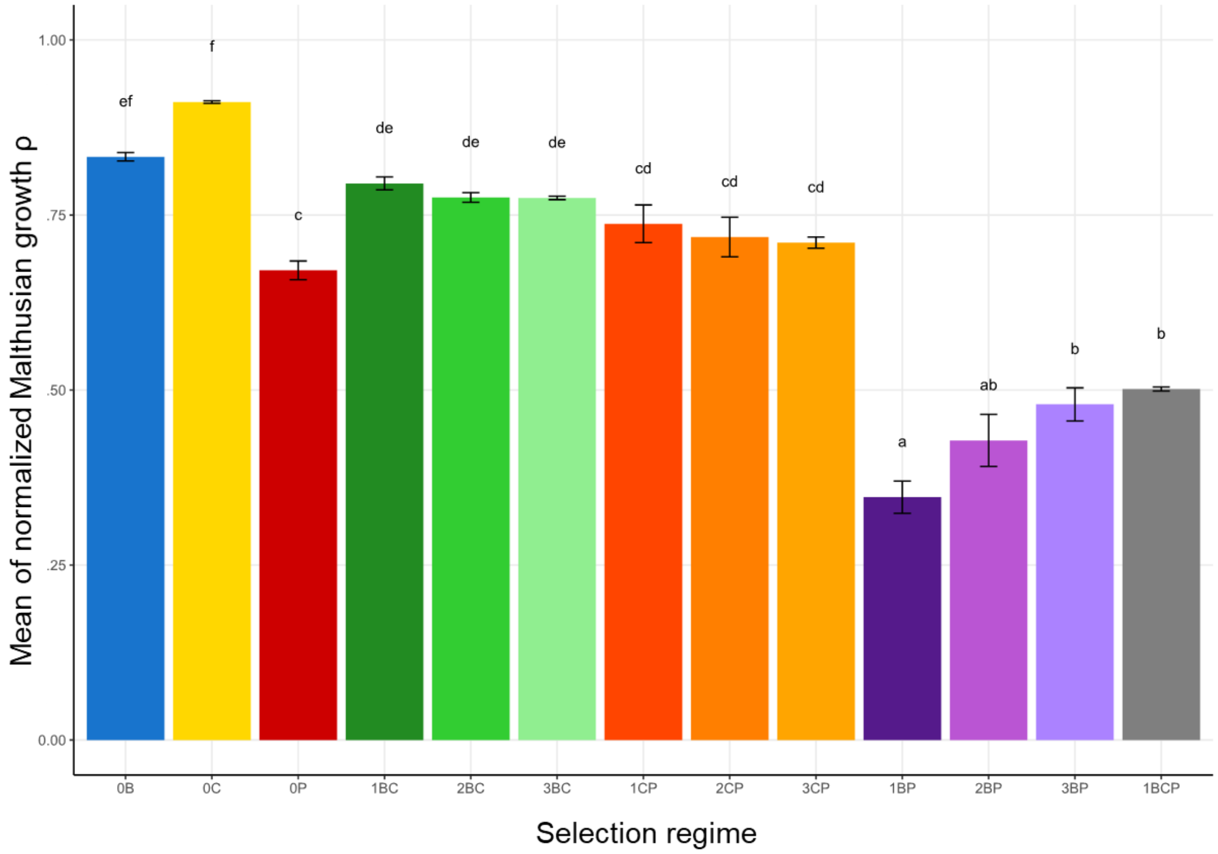

Supplementary Information Figure S2: Expression of *tub2*, *sdhC*, *cyp51* and *mfs1* in evolved strains of *Z. tritici*

Relative gene expression (expressed as $2^{-\Delta\Delta Ct}$) of *tub2*, *sdhC*, *cyp51*, encoding the target proteins of carbendazim, benzovindiflupyr and prothioconazole-desthio, respectively, shown for eight isolates collected from various selection regimes. The expression of the drug transporter MFS1 was also quantified, as enhanced efflux is recognized as a generalist resistance mechanism in field isolates of *Z. tritici.* Isolates were chosen to represent the diversity of sensitivity the various fungicides, as shown from their scores in droplet tests (shades of brown, on the top of each graph). The ancestral strain IPO-323 was added as negative control. Two biological replicates were tested independently. The expression of *tub2*, *cyp51* and *mfs1* was normalized to that of the genes encoding EF1α (black bars) and ubiquitin (light grey bars). The expression of *sdhC* was normalized to that of the gene encoding β-tubulin (grey bars).

The overexpression of the three target genes was not observed among isolates resistant to B, C or P. The overexpression of *msf1* is noticed for few isolates exhibiting moderate to high resistance to tolnaftate, suggesting that other unknown additional mechanisms must be involved.

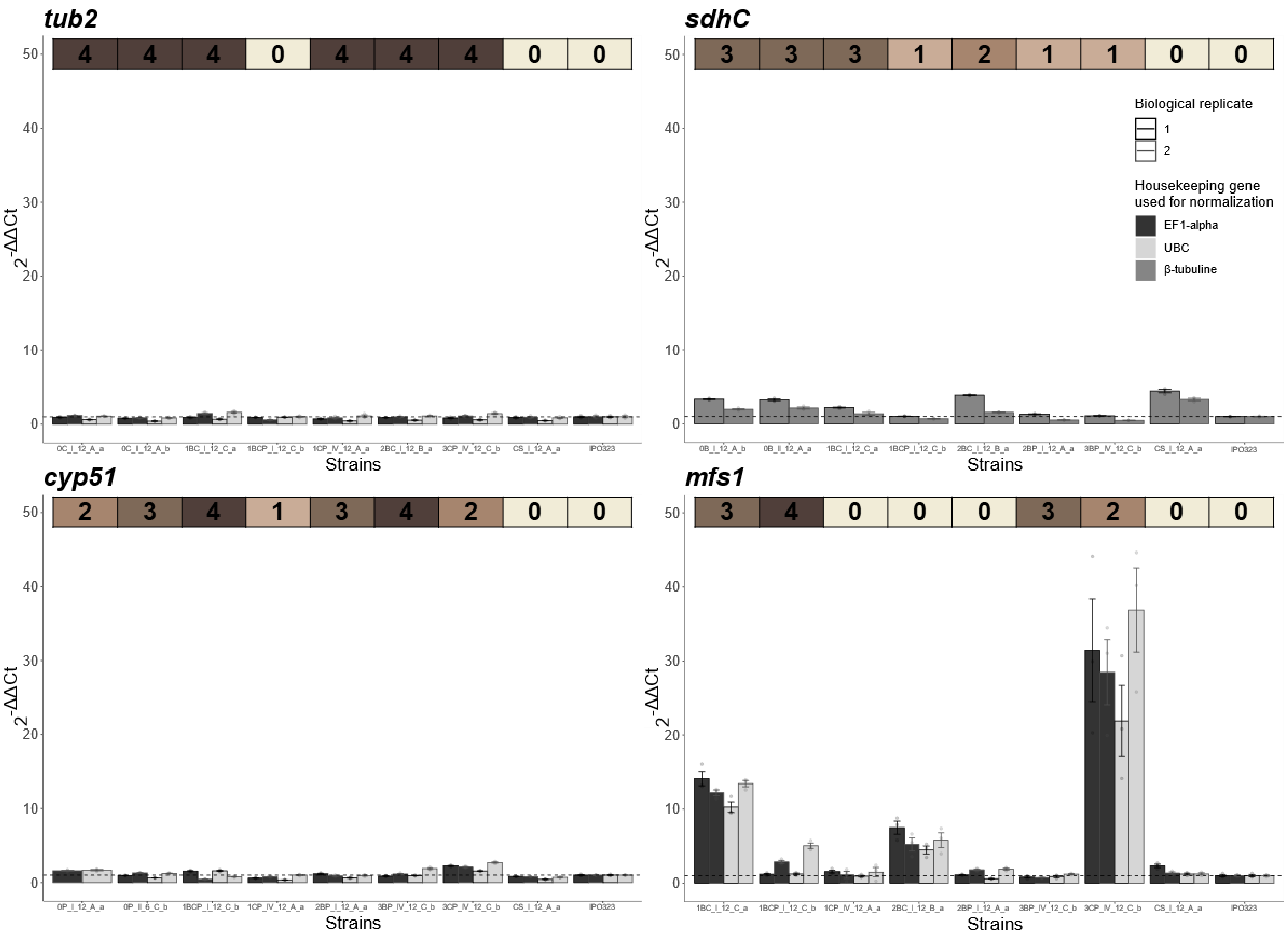

tub2
4 4 4 0 4 4 4 0 0
0C_I_12_A_a
0C_II_12_A_b
1BC_I_12_C_a
1BCP_I_12_C_b
1CP_IV_12_A_a
2BC_I_12_B_a
3CP_IV_12_C_b
CS_I_12_A_a
IPO323
sdhC
3 3 3 1 2 1 1 0 0
0B_I_12_A_b
0B_II_12_A_a
1BC_I_12_C_a
1BCP_I_12_C_b
2BC_I_12_B_a
2BP_I_12_A_a
3BP_IV_12_C_b
CS_I_12_A_a
IPO323
cyp51
2 3 4 1 3 4 2 0 0
0P_I_12_A_a
0P_II_6_C_b
1BCP_I_12_C_b
1CP_IV_12_A_a
2BP_I_12_A_a
3BP_IV_12_C_b
3CP_IV_12_C_b
CS_I_12_A_a
IPO323
mfs1
3 4 0 0 0 3 2 0 0
1BC_I_12_C_a
1BCP_I_12_C_b
1CP_IV_12_A_a
2BC_I_12_B_a
2BP_I_12_A_a
3BP_IV_12_C_b
3CP_IV_12_C_b
CS_I_12_A_a
IPO323
$2^{-\Delta\Delta Ct}$
Strains
0
10
20
30
40
50
Biological replicate
1
2
Housekeeping gene used for normalization
EF1-alpha
UBC
β-tubuline

Supplementary Information Methods S1: Statistical programs used for data analysis

All analyses were performed and all figures were generated with R 4.0.4 and R Studio 1.4.1106.
R Core Team. *R: A Language and Environment for Statistical Computing*. (R Foundation for Statistical Computing, 2021).
RStudio Team. *RStudio: Integrated Development Environment for R*. (RStudio, PBC, 2021).

The additional packages used for this work are listed below.

- **car**: Fox, J. & Weisberg, S. *An R Companion to Applied Regression*. (Sage, 2019).
- **cowplot:** Wilke, C. O. *cowplot: Streamlined Plot Theme and Plot Annotations for 'ggplot2'*. (2020).
- **coxed:** Kropko, J. & Harden, J. J. *coxed: Duration-Based Quantities of Interest for the Cox Proportional Hazards Model*. (2020).
- **emmeans**: Lenth, R. V. *emmeans: Estimated Marginal Means, aka Least-Squares Means*. (2021).
- **FactoMineR**: Lê, S., Josse, J. & Husson, F. FactoMineR: A Package for Multivariate Analysis. *Journal of Statistical Software* **25**, 1–18 (2008).
- **ggplot2:** Wickham, H. *ggplot2: Elegant Graphics for Data Analysis*. (Springer-Verlag New York, 2016).
- **ggpubr:** Kassambara, A. *ggpubr: 'ggplot2' Based Publication Ready Plots*. (2020).
- **ggdendro:** Vries, A. de & Ripley, B. D. *ggdendro: Create Dendrograms and Tree Diagrams Using 'ggplot2'*. (2020).
- **Multcomp:** Hothorn, T., Bretz, F. & Westfall, P. Simultaneous Inference in General Parametric Models. *Biometrical Journal* **50**, 346–363 (2008).
- **nlme:** Pinheiro, J., Bates, D., DebRoy, S., Sarkar, D., & R Core Team. *nlme: Linear and Nonlinear Mixed Effects Models*. (2021).
- **survival**: Therneau, T. M. *A Package for Survival Analysis in R*. (2020).

Supplementary Information Methods S2: Sequencing fungicide target genes in *Z. tritici*

1. **Primers used to sequence fungicide target genes and to detect alterations of the *MFS1* promoter in *Z. tritici***

**Primer name**	**Primer sequence**	**Tm** (°C)	**Reference**
Tub2			
Tub5	CAGTCACTTGCAAACGGGTA (F)	63.7	This work
Tub14	GAAGCCTCCTGGTACTGCTG (R)	63.9	-
Tub15	GTCGACCAGGTTCTCGATGT (F)	64.1	-
Tub13	AACCGACCATGAAGAAGTGG (R)	63.8	-
cyp51			
CypFra2r	TCCTTCTCCTCCCTCCTCTC (R)	64.0	Brunner *et al* (2008)
EBI112	TTCAGCACGCTCGCCATCCTCC (F)	76.5	Brunner *et al* (2008)
Cyp9	TCTATACTGCCAGCCGATCA (R)	63.3	This work
Cyp12	CACTAACAGGCCAACTGCAA (F)	63.8	-
NB1	CACTCTTCATCTGCGACCGAGTC (R)	69.4	Leroux *et al* (2007)
sdhB			
Mg SDHB	TTCGGCACGTCGAGTTTCTGGCCATGCTGGCGG (F)	89.2	This work
Mg SDHB Rv	CCACAATGCCGCAAAATCGGCACAACTGCTCCC (R)	86.1	-
Mg SeqSDHB Fw	ACATCTACCGATGGAACCC (F)	61.2	-
Mg SeqSDHB Rv	TGTGGCATCGGTACAAGC (R)	63.5	-
sdhC			
Mg SDHC Fw	TCTCTTCGTCGACATCACCA (F)	64.7	This work
Mg SDHC Rv	ACGCACTCGCAACACTCA (R)	64.7	-
sdhD			
Mg SDHD Fw	ACAGCTTCCGAGGTTTCGCG (F)	70.8	This work
Mg SDHD Rv	GCCATCTGTATATACACGCCC (R)	63.0	-

Brunner, P. C., et al. (2008). "Evolution of the CYP51 gene in *Mycosphaerella graminicola*: evidence for intragenic recombination and selective replacement." Molecular Plant Pathology **9**(3): 305-316.
Leroux, P., et al. (2007). "Mutations in the CYP51 gene correlated with changes in sensitivity to sterol 14 alpha-demethylation inhibitors in field isolates of *Mycosphaerella graminicola*." Pest Management Science **63**(7): 688-698.

2. **Regular PCR Mix**

Mix	1 tube (µL)
DNA	1
dNTP (25mM)	0.5
FW (10 µM)	1
RV (10 µM)	1
Titanium buffer (10X)	5

Titanium *Taq* polymerase (50X)	0.5
Deionized H_2O	41
Total volume	50

DNA polymerase: Titanium *Taq* (Takara Bio)

3. Regular PCR program

Initiation	95°C	3 min	
Denaturation	95°C	30 s	35 cycles
Annealing	Tm°C	30 s	35 cycles
Elongation	72°C	1min	35 cycles
Final	72°C	5 min	
	10°C	To the end of the cycle	

Supplementary Information Methods S3: Detecting polymorphism in promoters of target genes in *Z. tritici*

1. **Primers used to detect length polymorphism in the promotor sequence of fungicide target genes in *Z. tritici***

Primer name	Primer sequence	Tm (°C)	Reference
tub2			
Zt_tub2_prom_F1<br>Zt_tub2_prom_R1	ATTGCTGTGCTTGTGTTCGA (F)<br>CGACATACCCGTTTGCAAGT (R)	55.2<br>57.3	This work<br>-
cyp51			
Mg51proF<br>MgCyp51-5'-1r	GTGGCGAGGGCTTGACTAC (F)<br>CAAGTTTCCAGAGGCTGGTC (R)	61.0<br>59.4	Chassot *et al,* 2008<br>-
sdhC			
Zt_sdhC_prom_F1<br>Zt_sdhC_prom_R1	GACCGGTCGAATGCTTGC (F)<br>GGTGAGCTTCTGTGCCAAC (R)	58.2<br>58.8	This work<br>-
mfs1			
MFS1_consensus_2<br>MFS1_consensus_4	GCAAGGATTCGGACTTGACG (F)<br>CTGCCGGTATCGTCGATGAC (R)	66.9<br>67.8	Omrane *et al,* 2017<br>**-**

Chassot, C., et al. (2008). Sensitivity of *Cyp51* genotypes to DMI fungicides in *Mycosphaerella graminicola*. Modern Fungicides and Antifungals compounds V. G. U. Dehne DW, Kuck KH, Russell PE and Lyr H. Braunschweig, Germany, DPG Seebsterverlag**:** 129-136.
Omrane, S., et al. (2017). "Plasticity of the *MFS1* promoter leads to multidrug resistance in the wheat pathogen *Zymoseptoria tritici*." mSphere **2**(5).

2. **Regular PCR Mix**

Mix	1 tube (µL)
DNA	1
dNTP (25mM)	0.24
Primer FW (10 µM)	0.5
Primer RV (10 µM)	0.5
Titanium buffer (10X)	2.5
Titanium *Taq* polymerase (50X)	0.3
Deionized H_2O	19.96
Total volume	25

DNA polymerase: Titanium *Taq* (Takara Bio)

3. Regular PCR program

For *cyp51, sdhC* and *mfs1*

Initiation	95°C	3 min	
Denaturation	95°C	30 s	35 cycles
Annealing	Tm°C	30 s	
Elongation	72°C	1min	
Final	72°C	5 min	
	10°C	To the end of the cycle	

For *tub2*

Initiation	95°C	3 min	
Denaturation	95°C	30 s	15cycles
	68°C	1mn	
Denaturation	95°C	30 s	35 cycles
Annealing	Tm°C	30 s	
Elongation	72°C	1min	
Final	72°C	5 min	

Supplementary Information Methods S4: Quantifying the expression of *tub2*, *cyp51*, *sdhC*, *mfs1* in *Z. tritici* by qRT-PCR

1. Origin of tested isolates

Isolate	Selection regime	Tested genes
IPO323	None (ancestral strain)	*tub2*, *cyp51*, *sdhC* and *mfs1*
CS_I_12_A_a	Control with 0.5% ethanol as solvent	*tub2*, *cyp51*, *sdhC* and *mfs1*
0C_I_12_A_a	0C	*tub2*
0C_II_12_A_b	0C	*tub2*
0B_I_12_A_b	0B	*sdhC*
0B_II_12_A_a	0B	*sdhC*
0P_I_12_A_a	0P	*cyp51*
0P_II_6_C_b	0P	*cyp51*
1CP_IV_12_A_a	1CP	*tub2, cyp51* and *mfs1*
3CP_IV_12_C_b	3CP	*tub2, cyp51* and *mfs1*
1BC_I_12_C_a	1BC	*tub2, sdhC* and *mfs1*
2BC_I_12_B_a	2BC	*tub2, sdhC* and *mfs1*
2BP_I_12_A_a	2BP	*cyp51, sdhC* and *mfs1*
3BP_IV_12_C_b	3BP	*cyp51, sdhC* and *mfs1*
1BCP_I_12_C_b	1BCP	*tub2, cyp51, sdhC* and *mfs1*

2. Primers used in qPCR

Primer name	Primer sequence (5'-3')	Reference	Use
β-tubulin_Fw<br>β-tubulin_Rv	AACGAGGCTCTCTACGACATCTG<br>GGCGGAGACGAGGTGGTTG	Omrane *et al*, 2017	Expression of *tub2*, encoding β-tubulin
CYP51_Fw<br>CYP51_Rv	TTCTCTTCCGTGGCAAGTTGTC<br>GCCGTATGTGATGGTGCTTCC	S. Patry-Leclaire, unpublished	Expression of *cyp51*, encoding sterol 14α-demethylase
MFS1_F<br>MFS1_R	CAATGGGGCGGCAGCAAATAC<br>TTGGATGGTGGCGAAGATGAGG	Omrane *et al*, 2015	Expression of *mfs1,* encoding a MFS drug transporter
UBC_Fw<br>UBC_RV	GTCTGCGGACCACAATACC<br>CGACCTTTCCTTGCCTCTG	Omrane *et al*, 2017	Ubiquitine housekeeping gene

EF1α_Fw	AAGATTGGTGGTATCGGAACAG	Omrane *et al*, 2017	Elongation factor EF1α housekeeping gene
EF1α_Rv	GACTTGACTTCGGTGGTGAC		
qPCR_SDHC1_fwd	GGCAACAACTCCTTCCAACAG	Steinhauer *et al,* 2019	Expression of *sdhC*, encoding the C subunit of the succinate dehydrogenase
qPCR_SDHC1_rev<br>qPCR_SDHC1_probe (CY5/BHQ2)	ACCAGGTTATTTGCGGTTTGT<br>CCAAGTAACAGCAGCCGCCGTCTCCGAATC		
qPCR_TUB1_fwd<br>qPCR_TUB1_rev<br>qPCR_TUB1_probe (HEX / BHQ1)	AGCGCATGAATGTCTACTTCAA<br>CCTTGGCCCAGTTGTTTCC<br>TCGGTCAGCTCTTCCGCCCAGA	Steinhauer *et al,* 2019	β-tubulin housekeeping gene (for *sdhC* only)

EF1α and *UBC* were used as reference genes to quantify the expression of *tub2, cyp51* and *mfs1. tub2* was used as a reference gene to quantify the expression of *sdhc.*

Steinhauer, D., et al. (2019). "A dispensable paralog of succinate dehydrogenase subunit C mediates standing resistance towards a subclass of SDHI fungicides in *Zymoseptoria tritici*." PLOS Pathogens **15**(12): e1007780.Omrane, S., et al. (2015). "Fungicide efflux and the MgMFS1 transporter contribute to the multidrug resistance phenotype in *Zymoseptoria tritici* field isolates." Environmental Microbiology **17**(8): 2805-2823.
Omrane, S., et al. (2017). "Plasticity of the *MFS1* promoter leads to multidrug resistance in the wheat pathogen *Zymoseptoria tritici*." mSphere **2**(5).

3. Protocols

Isolates were grown in the dark at 18°C, with a RH of 70% and shaking at 150 rpm for four days, in 50 mL borosilicate Erlenmeyer flasks containing 25 mL YPD medium and no fungicide. Two independent biological repeats, grown successively, were produced for each isolate. Fungal cells were freeze-dried and RNAs were extracted using a commercial kit (Monarch total RNA Miniprep; New England Biolabs) and the manufacturer's recommendations. Retrotranscription was achieved using the protoScript II First Strand cDNA Synthesis kit (New England BioLabs) according to the manufacturer's protocol. qPCRs were achieved as follows, with three technical repeats for each sample:

Mix	1 tube (µL)
cDNA	2
Primer FW (10 µM)	0.4
Primer RV (10 µM)	0.4
Master Mix (2X)	10
Deionized H_2O	7.2
Total volume	20

DNA polymerase: MESA GREEN qPCR MasterMix Plus for SYBR Assay (Eurogentec)

Regular qPCR program for *tub2, cyp51, mfs1, EF1α* and *UBC*

95°C	5 min	
95°C	15 s	45 cycles
60°C	1min	

Regular qPCR program for *sdhC*

95°C	3 min	
95°C	10 s	45 cycles
60°C	20 s	

4. Determination of the relative gene expression, $2^{-\Delta\Delta Ct}$

First, ΔCt values were calculated as:

$$\Delta \mathrm{Ct} = Ct_{\mathrm{i,\ target\ gene}} - Ct_{\mathrm{i,\ housekeeping\ gene}}$$

with Ct the value of cycle threshold and i each isolate tested.

ΔΔCt values were calculated after the normalization from the control ancestral strain IPO323:

$$\Delta\Delta \mathrm{Ct} = \Delta \mathrm{Ct_i} - \Delta \mathrm{Ct_{IPO323}}$$
